# Effects of technology-based educational tools on nursing learning outcomes in intensive care units: a systematic review and meta-analysis

**DOI:** 10.1186/s12909-022-03810-z

**Published:** 2022-12-03

**Authors:** Sorayya Rezayi, Alireza Amanollahi, Leila Shahmoradi, Nafiseh Rezaei, Meysam Rahmani Katigari, Mitra Zolfaghari, Babak Manafi

**Affiliations:** 1 Health Information Management and Medical Informatics Department, School of Allied Medical Sciences, Tehran University of Medical Sciences, Tehran, Iran; 2 Department of Epidemiology, School of Public Health and Safety, Shahid Beheshti University of Medical Sciences, Tehran, Iran; 3 Trauma and Injury Research Center, Iran University of Medical Sciences, Tehran, Iran; 4 Department of Medical Library & Information Science, School of Paramedicine, Hamadan University of Medical Sciences, Hamadan, Iran; 5 Medical Library and Information Science, School of Allied Medical Sciences, Tehran University of Medical Sciences, Tehran, Iran; 6 Health Information Technology Department, Saveh University of Medical Sciences, Saveh, Iran; 7 Nursing and Midwifery Care Research Center, School of Nursing and Midwifery, Tehran University of Medical Sciences, Tehran, Iran; 8 Department of Heart Surgery, School of Medicine, Hamadan University of Medical Sciences, Hamadan, Iran

**Keywords:** Technology, Nursing education, Intensive care, Critical care, Electronic learning, Systematic review, Meta-analysis

## Abstract

**Background:**

Nurses working in the Intensive Care Unit (ICU), due to the sensitivity and difficulty of tasks, need continuous and scientific training to be able to offer the best performance in difficult situations and use their knowledge in the best way. Also, nursing students spend internships in ICUs and receive special training in practice in the actual center. Educational tools based on new technologies can potentially improve the educational outcomes of nursing in ICUs.

**Objectives:**

The present study aims to review and evaluate the effect of using technology-based educational tools for training critical care nurses and nursing students.

**Methods:**

A comprehensive search was conducted to identify peer-reviewed English language articles in Embase, Medline (through PubMed), Scopus, and ISI web of science published from 2010 to Feb 18, 2022. The studies that examined the effectiveness of technology-based educational interventions with control groups were included. The risk of bias in each study was assessed using the Cochrane Collaboration’s tool. Also, we used Standard Mean Difference (SMD) to estimate the effect of technology-based educational tools on learning outcomes. All meta-analyses were performed with a random effects model in Stata Ver.16.

**Results:**

Altogether, ten studies were eligible for the quality assessment and systematic review, while one study that had not reported the pre-intervention analysis was excluded from the meta-analysis. Nine studies were considered to have a low RoB regarding reporting ways, and one of them showed a high risk. Performance and selection bias caused a high risk in six and five of the studies, respectively. In the meta-analysis, improvement in knowledge (SMD = 0.91), skills (SMD = 0.52), and self-confidence (SMD = 0.96) was noticed by applying technology-based educational tools.

**Conclusion:**

It can be offered that if the learning method based on the new technologies tested is more effective than conventional teaching methods, they are likely to improve the learning outcome significantly. The new-developed tools also have great potential in improving health care functions among nurses or nursing students as well as enhancing the quality of life and patient satisfaction.

**Supplementary Information:**

The online version contains supplementary material available at 10.1186/s12909-022-03810-z.

## What is already known

- Nursing care for critically ill patients includes essential care tasks that support the promotion of patients' health or the maintenance of their clinical condition.
- These various educational tools, for further development and general use, should be well introduced and their impact on multiple applications should be evaluated.

## What this paper adds

- In the meta-analysis, improvement in knowledge (SMD = 0.91), skills (SMD = 0.52), and self-confidence (SMD = 0.96) effect size was noticed in the technology-based educational tools.
- This study explained that technology-based training solutions such as virtual reality, simulation-based e-learning, social networks, etc., have significant potential to improve outcomes.

## Introduction

Nurses, as one of the most important providers of health services, play an essential role in the persistence of health care, and promotion at different levels of health services. To maintain patient safety, nursing managers should provide appropriate training protocols to improve the knowledge of nurses. Lee and Chang [1] stated that nursing involves four different levels of professional competence, and critical care units nurses must have the highest professional level of nursing. The services and care provided in this unit require higher vigilance and quality than other units [2]. Patients in critical care units often experience multiple organ dysfunction, hemodynamic instability, complex medication regimens, as well as vulnerability to stress for both themselves and their families. Nursing care for critically ill patients includes essential care tasks that support the improvement of patients' health or the maintenance of their clinical condition [3, 4].

Most patients in this unit are usually under mechanical ventilation and are unconscious or in a coma. Thus, providing care to patients requires nurses who are equipped with up-to-date knowledge, alertness, and expertise [5]. The conditions of patients in critical care units are variable and very complex, and nurses in these wards need to be able to assess and provide care for critically ill patients, evaluating symptoms, and intervene with initial assessments as well as treatment to avoid unexpected risks [6]. In critical care, the nurse constantly encounters patients whose health status changes rapidly. These require quick decisions in a short time despite massive stress [7]. Research shows that there is a clear correlation between nurse skills and patient outcomes. The complexity of the role of nurses in these units requires a structured and continuous training program from elementary to graduate courses [8]. Nurses or nursing students learn through ongoing training to quickly identify problems or abnormalities in patients by evaluating data, influential factors, and potential health risk factors [9].

Until now, various nursing educations in university or in-service courses have been presented in a traditional way and as a lecture. However, this approach has its own problems. For example, not only does it require more human, financial, and equipment resources, but also people have to leave their work environment to participate in the course. Thus, traditional teaching methods should be changed to improve learning experiences and facilitate lifelong learning. Teaching strategies that include hands-on experience through doing and communicating as well as talking with others promote more meaningful learning. This approach will develop creativity and innovation for both students and teachers [10].

The unique capability of information technology has provided the possibility that soon, educational systems, as well as other areas, will be ultimately affected [11]. Currently, many universities around the world use information technology to develop and improve medical education [12]. In 1986, computers were predicted to become an inevitable part of the medical education system [13]. In 2000, nursing education strategies expanded from simple online reading courses to learning through various mobile devices as well as interactive learning with peers and educators [14]. Now, with the implementation of social distancing protocols to contain the spread of the COVID-19 disease, the use of technology in education and learning has peaked [15]. While the traditional lecture style focuses on face-to-face lectures, making the learner a passive participant, technology-based learning has emerged as a new learner-centered teaching method which facilitates learner participation and feedback [16]. Studies have shown that the electronic learning method has been successful in teaching various fields of nursing concepts and skills, such as drug calculations, arterial blood gas interpretation, electrocardiogram interpretation, control of vital signs, triage, report writing, the correct method of hand washing as well as many other cases [17–19]. It has also changed the level of knowledge, behavior, and performance of nurses and nursing students. Learning management systems worked so well that nursing courses began migrating online. Currently, many educators use simulators to teach students how to diagnose heart problems [20]. The Cardiopulmonary Resuscitation (CPR) Training System of the American Heart Association is also one of the most widely used useful educational tools. Other programs such as drug management, anatomy, physiology are also used [21]. Nursing simulation tools, augmented reality and virtual reality have also recently become popular and are used to stimulate motivation and improve learning [14].

### Aim of the study

For further development and general use, various educational tools should be well introduced and their impact on various applications should be evaluated. Accordingly, the present study aims to review, synthesize, and analyze the effects of technology-based educational tools for training critical care nurses and nursing students. Meta-analysis was also used to examine this impact on nurses’ or nursing students' skills, knowledge, self-confidence, and attention. It helps summarize different scientific documents and summarize them in an objective way with minimum personal opinions.

## Methods and materials

This systematic review and meta-analysis were conducted based on the Preferred Reporting Items for Systematic Review and Meta-analysis (PRISMA) tool proposed by Matthew J Page et al. [22]. PRISMA is an evidence-based minimum set of items for reporting in systematic reviews and meta-analyses. PRISMA primarily focuses on the reporting of reviews evaluating the effects of interventions, though it can also be used as a basis for reporting systematic reviews with objectives other than evaluating interventions [23]. The filled PRISMA checklist is given as supplementary material (Appendix Table 1). Also, this review was conducted in line with the Cochrane Handbook for Systematic Reviews and Interventions but no protocol was registered [24]. We applied the quantitative and qualitative analysis process to summarize the screened papers and generate new notable insights.

### Data sources and search strategy

Medline (through PubMed), EMBASE, Scopus, and Web of Sciences (WOS) were selected as core search databases. The mentioned databases were selected because of their coverage of qualitative and health research. We identified papers with a time limit, where articles published from 2010 to 18 Feb 2022, were examined. The search strategy used in this study involved a combination of keywords and Medical Subject Headings (Mesh) terms and Emtree related to “nursing”, “Education”, “technology”, “Computer education”, and “Intensive care”. The Emtree thesaurus is a hierarchically structured, controlled vocabulary for biomedicine and the related life sciences. It includes a whole range of terms for drugs, diseases, medical devices and essential life science concepts. Emtree is employed to index all of the Embase content. Hence, Emtree is the collection of standardized keywords in Embase. The use of standard keywords for each concept leads to the formulation of a complete search strategy. The complete list of keywords applied in the search strategy is provided in Table 1. Reference manager software (EndNote X8, Thomson Reuters) was utilized to collect references and exclude duplicates.

**Table 1:** Search strategy for all databases

**Database**	**Search strategy**
**PubMed**	( nurs*[TIAB] OR "Nurses"[Mesh] OR "Education, Nursing"[Mesh]) AND ( video [TIAB] OR online[TIAB] OR on-line[TIAB] OR virtual[TIAB] OR elearning[TIAB] OR e-learning[TIAB] OR "Augmented reality"[TIAB] OR tele*[TIAB] OR electronic [TIAB] OR Eeducation[TIAB] OR e-education[TIAB] OR internet [TIAB] OR mobile[TIAB] OR web*[TIAB]) AND ( technolog*[TIAB] OR tool* [TIAB] OR application*[TIAB] OR software* [TIAB] OR hardware*[TIAB] OR program*[TIAB] OR booklet*[TIAB] OR app[TIAB]) AND ("Education"[Mesh] OR educat*[TIAB] OR learn*[TIAB] OR train*[TIAB] OR teach* [TIAB]) AND ( "Intensive Care" [TIAB] OR "Critical Care"[TIAB] OR "Critical Care"[Mesh] OR ICU [TIAB] OR "Intensive Care Units"[Mesh]) Time limitation: 2010–2022
**Scopus**	TITLE-ABS-KEY( nurs* AND ( video OR online OR on-line OR virtual OR elearning OR e-learning OR "Augmented reality" OR tele* OR electronic OR Eeducation OR e-education OR internet OR mobile OR web*) AND ( technolog* OR tool* OR application* OR software* OR hardware* OR program* OR booklet* OR app) AND ( educat* OR learn* OR train* OR teach*) AND ( "Intensive Care" OR "Critical Care" OR ICU)) Time limitation: 2010–2022
**WOS**	TS = ( nurs* AND ( video OR online OR on-line OR virtual OR elearning OR e-learning OR "Augmented reality" OR tele* OR electronic OR Eeducation OR e-education OR internet OR mobile OR web*) AND ( technolog* OR tool* OR application* OR software* OR hardware* OR program* OR booklet* OR app) AND ( educat* OR learn* OR train* OR teach*) AND ( "Intensive Care" OR "Critical Care" OR ICU)) Time limitation: 2010–2022
**Embase**	1. nurs*.ab. or nurs*.ti. or nurs*.kw 2. exp nurse/ 3. 1 or 2 (video or online or on-line or virtual or elearning or e-learning or "Augmented reality" or tele* or electronic or Eeducation or e-education or internet or mobile or web*).ab. or (video or online or on-line or virtual or elearning or e-learning or "Augmented reality" or tele* or electronic or Eeducation or e-education or internet or mobile or web*).ti. or (video or online or on-line or virtual or elearning or e-learning or "Augmented reality" or tele* or electronic or Eeducation or e-education or internet or mobile or web*).kw 4. (technolog* or tool* or application* or software* or hardware* or program* or booklet* or app).ab. or (technolog* or tool* or application* or software* or hardware* or program* or booklet* or app).ti. or (technolog* or tool* or application* or software* or hardware* or program* or booklet* or app).kw (educat* or learn* or train* or teach*).ab. or (educat* or learn* or train* or teach*).ti. or (educat* or learn* or train* or teach*).kw 5. exp education/ 6. (educat* or learn* or train* or teach*).ab. or (educat* or learn* or train* or teach*).ti. or (educat* or learn* or train* or teach*).kw 7. exp education/ 8. 6 or 7 9. ("Intensive Care" or "Critical Care" or ICU).ab. or ("Intensive Care" or "Critical Care" or ICU).ti. or ("Intensive Care" or "Critical Care" or ICU).kw 10. exp intensive care/ Time limitation: 2010–2022

### Selection criteria

#### Inclusion criteria

**Fig. 1:**
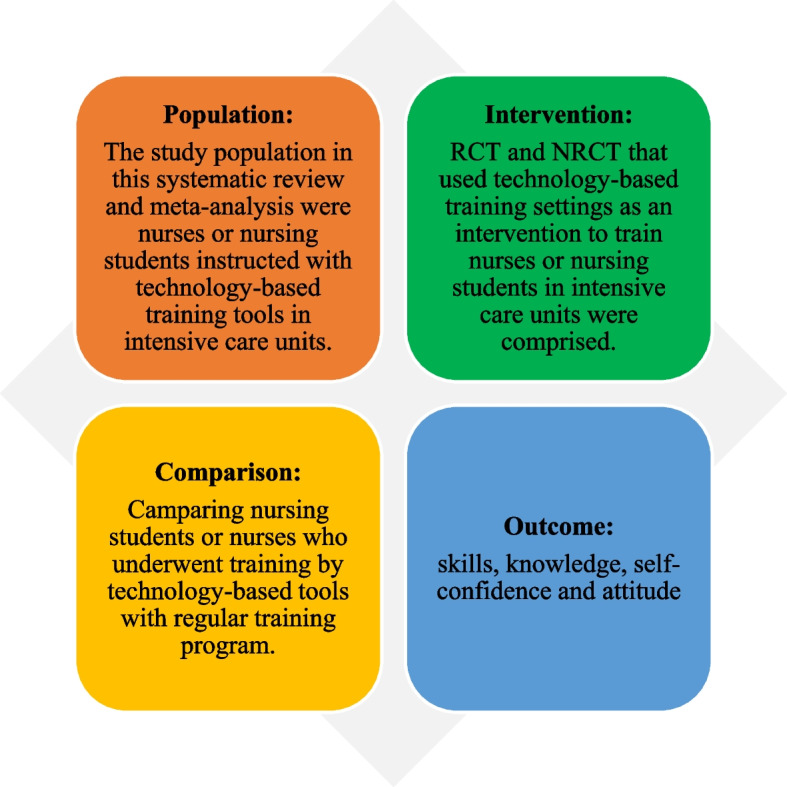
Inclusion criteria based on PICO in this SR and meta-analysis

The inclusion criteria set, which were admitted in this systematic review (SR) and meta-analysis, are outlined in Fig. 1. Types of studies in this review included Randomized Clinical Trial (RCT) and Non-Randomized Clinical Trial (NRCT). Accordingly, the PICO model was selected for this purpose. This means a reliable and comprehensive question should comprise four parts that recognize the patient problem or Population (P), Intervention (I), Comparison (C), and Outcome(s) (O).

#### Exclusion criteria

Articles were excluded if they were:

- (1) observational studies or non-experimental studies,
- (2) conference papers,
- (3) studies in which technology-based educational tools were excluded,
- (4) studies in which the target group were not nursing students or nurses were excluded,
- (5) non-English papers.

### Study selection

In this stage, the assessment of studies was done by more than one reviewer. Two reviewers independently screened the titles and abstracts of the identified studies. Any disagreement between the reviewers was resolved through discussion with a third researcher. The full text of the citations was retrieved and examined if it was supposed potentially relevant by two reviewers too.

### Data extraction

Here, the main classification of reviewed articles was determined. Three authors synthesized and analyzed the main specifications of included papers, after which they extracted the main key items of the papers. The authors assessed the extracted information and validated the main elements. If there was any discrepancy in the extracted data, a consensus was reached with thorough discussion after repeating the same extraction process. General characteristics such as authors, publication year, country, journal, education domain, population plus sample size (with a mean age of participants), study design, technology-based intervention, setting, sessions details (number, duration, and frequency), measurement time point, follow-up duration, learning outcomes, evaluation results, main message, and reported limitations were extracted from individual studies. Before extracting the data from the full texts of the articles, an interrater reliability check between the evaluators was performed. At this stage, 50% of the included articles and 20% of the excluded articles were randomly selected by two authors, and interrater reliability checks were performed. There was no disagreement between the authors. The following data were extracted from the selected studies and entered into a structured form in Excel.

### Study risk of bias assessment

To assess the risk of bias in individual included papers, the Cochrane Collaboration’s risk of bias tool was applied. Studies with a high or unclear risk of bias for the blinding of assessors or incomplete outcome data categories were considered as high risk of bias [25]. This tool addressed external validity, internal validity, and interpretability. Assessments were conducted by three independent authors. Two reviewers established consensus scores and resolved disagreements.

### Evidence synthesis and analyses

We imputed the mean changes (before, after) and the pooled standard deviation of the study outcome values in the meta-analysis. All analyses related to meta-analysis were performed using a model with random effects. Estimated values for pooled effect sizes in all learning outcomes were shown with Standardized Mean Differences (SMD); this method was chosen due to the different parameter scales in the selected studies. Heterogeneity was assessed with I^2^, τ^2^ tests, while publication bias was evaluated by Egger’s, Begg’s test. All analyses were conducted in Stata v16 and EndNote X9 was applied for resource management. Hence, Mean Gain (MG) and SD Pooled from pre and post-intervention were inputted into Stata v16.

## Results

**Fig. 2:**
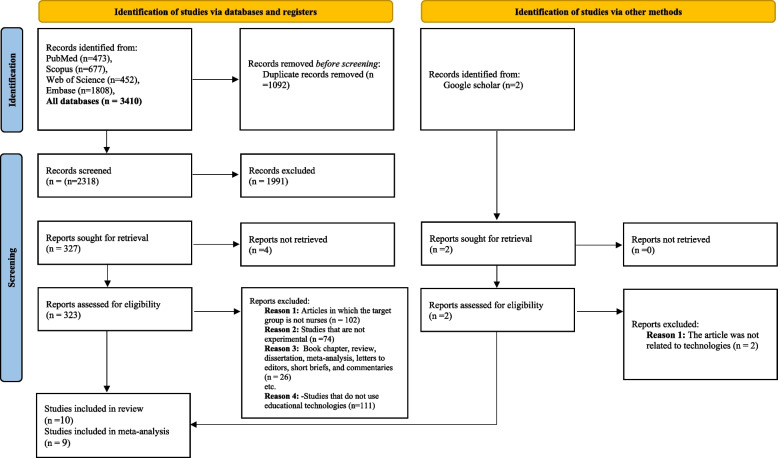
The PRISMA diagram for the search of records and study selection

A total of 3410 relevant articles were resulted from the search from 2010 until Feb 2022. After the removal of duplicates, 2318 articles remained. The process of searching the four main databases and identifying studies based on the PRISMA diagram is displayed in Fig. 2. Title and abstract screening led to the omission of 1991 articles. In the first examination, 323 papers seemed relevant, and their full text was investigated. After examining the full text of the identified papers and applying the inclusion plus exclusion criteria, ten studies were included in this systematic review and nine of them were synthesized in meta-analysis.

### Risk of bias (RoB) in the included studies

**Fig. 3:**
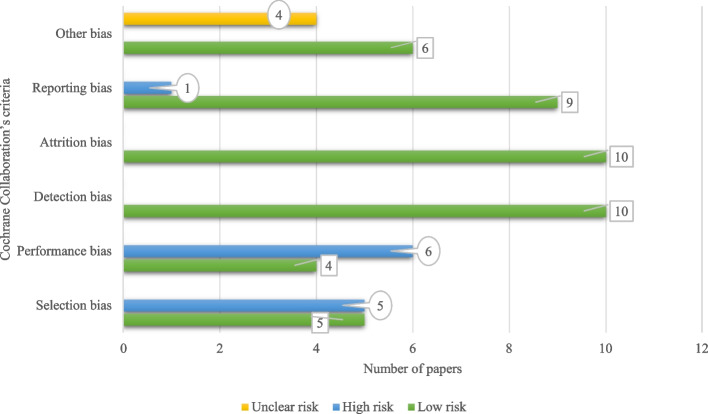
Cochrane Collaboration’s tool for assessing the risk of bias in papers

All articles have the lowest bias value regarding detection and attrition aspects. Four studies were assessed to have an unclear RoB for any crucial concerns about bias not covered in the other domains in the tool. Nine papers were considered to have a low RoB in reporting ways, and one of them had a high risk. Totally, based on results, nine papers were assessed as good quality studies, though not without risk of bias. Qualitative assessment for all the individual papers is shown in Fig. 3. Remarkably, selection bias and performance bias refer to “biased allocation to interventions due to inadequate generation of a randomized” and “bias due to knowledge of the allocated interventions by participants and personnel during the study” were assessed for some studies with high risk. For performance bias, two aspects have been considered in the studies: blinding participants and blinding personnel or researchers. In studies with high bias risk, neither participants nor personnel were blinded, whereas, in studies with low risk, participants were not blinded, but staff and assessors were blinded.

### General characteristics of the included studies

**Fig. 4:**
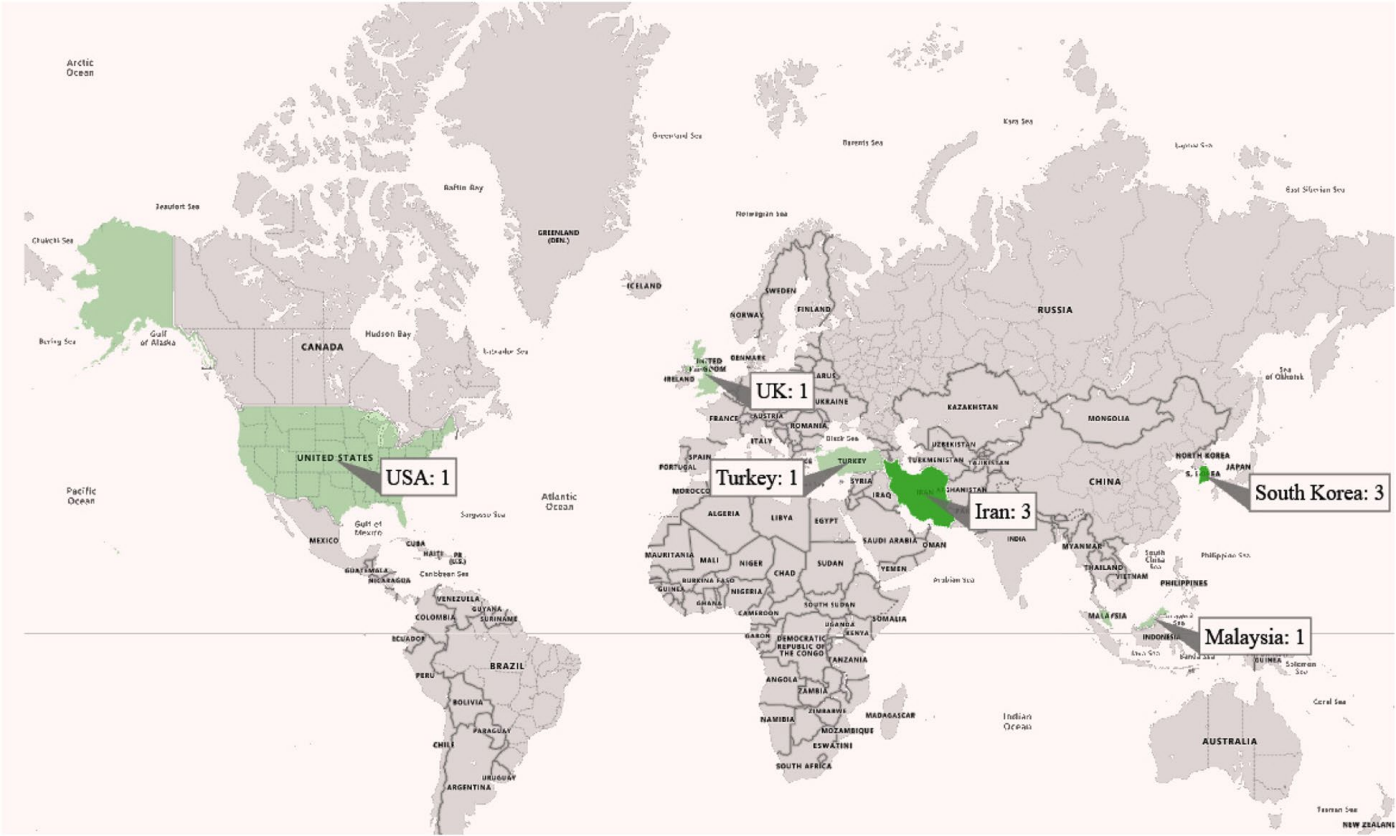
The distribution of articles based on countries

Table 2 presents the general characteristics of the included studies. The oldest and newest articles were published in 2016 and 2022, respectively. For most investigations, 60% (6/10) of papers were conducted in South Korea and Iran; the distribution of papers based on countries is presented in Fig. 4. In the screened studies, 737 nurses and nursing students participated; 367 participants were in the experimental groups (EG), and 366 were in the control groups (CG). Eight studies reported the mean age of participants; the range of mean age was 21.18 years old to 34 years old. The sample size ranged from 44 to 122 participants (IQR1:47, median: 68, IQR3: 100). In seven studies, the participants were ward nurses, and in three studies, nursing students were trained. However, the number of intervention sessions was not homogeneous and, in some experimental ones, was not mentioned clearly.

**Table 2:** General characteristics of the included studies (*n* = 10)

**Contribution**	**Journal**	**Education domain**	**Population and sample size (Mean age)**	**Study design**	**Technology-based intervention**	**Setting**	**Sessions details (number, duration and frequency)**	**Measurement time point**	**Intervention group**	**Control group**	**Follow up duration**	**Learning outcomes**	**Evaluation result**	**Main message**
Yang,2021, South Korea [26]	International Journal of Environmental Research and Public Health	End-of-life care education in the ICU	44 nurses, Intervention group: *n* = 23 (25.14 ± 2.27) Control group: *n* = 21 (27.14 ± 2.52)	Quasi-experimental design	End-of-Life Care Mobile App (EOL Care App)	ICU	7 ses, each ses 30 min, till 7 days	Baseline and after intervention	EOL app download and installation on the mobile phone	End-of-life care booklet	Without follow up	knowledge, self-efficacy, and compassion	knowledge (0.44 ± 0.19 to 0.76 ± 0.14, *p* < 0.001); self-efficacy (*p* < = 0.001); compassion (to 0.73 ± 0.49, *p* = 0.005)	According to the results, the end-of-life care mobile app was an effective educational method for nurses with experience of less than 3 years in an intensive-care unit
Moon,2019, South Korea [27]	BMC Medical Education	Cardiopulmonary resuscitation education	120 nursing students, Intervention group: *n* = 60 (21.18 ± 1.08) Control group: *n* = 60 (21.27 ± 1.16)	RCT	CPR e-learning program	CICU	4 ses, 60 min, 30 min, 50 min, 90 min	Baseline and after intervention	Blended learning CPR education program	The printout containing CPR guidelines	Without follow up	knowledge, attitude, and self-efficacy	knowledge (intervention: 16.40 ± 1.56, control: 6.46 ± 2, *p* < .001), and emotional attitude (intervention: 40.85 ± 8.01, control: 36.05 ± 6.87, *p* = .002), self-efficacy (no significant differences)	Learning CPR program was found effective in improving nursing students’ knowledge and attitudes regarding CPR
Starodub,2020, USA [28]	International Emergency Nursing	Targeted temperature management (TTM) for cardiac arrest patients	52 nurses (33.6 ± 9.5) years), Intervention group: 24 Control group: 28	A clustered RCT	Video lecture with high fidelity simulation	CICU	Not mentioned	Baseline, after intervention and after follow up duration	Video lecture with high fidelity simulation	Video lecture	2 and 6 weeks	knowledge, skills, confidence and satisfaction	knowledge was significant (beta = 7.93, SE = 3.88, *p* = .04), psychomotor skills was significant (beta = 11.77, SE = 4.12, *p* = .004), no significant difference in confidence after the intervention, t(50) = − 0.92, *p* = .36, in the simulation group were more satisfied, *p* = .0002	Nurses trained with video lecture and high-fidelity simulation benefitted from this approach by maintaining their TTM knowledge longer
Deldar,2020, Iran [29]	BMC Medical Education	Observational pain diagnosis and assessment in mechanically-ventilated patients	70 nurses, Interventional group: 34 (34 ± 7) Control group: 33 (34 ± 6)	Quasi-experimental design	Social networking app	ICU	Every day by 2 weeks	Baseline and after intervention	Training with Social networking app	Routine lectures	2 weeks	Performance	Mean score of pain diagnosis (82 ± 19 in the lecture group) and (97 ± 8 in the social networking app group (*P* < 0.01)), Mean pain management scores (30 ± 17 and 90 ± 18 (*P* < 0.01))	learning through a social networking app led to improved diagnosis and management of pain in mechanically-ventilated patients
Najafi Ghezeljeh,2022, Iran [30]	Journal of the Intensive Care Society	Delirium recognition ability and delirium-related strain of care	88 nurses, Intervention group: 43 (30.88 ± 4.90) Control group: 45 (31.40 ± 5.71)	Quasi-experimental design	E-learning	ICU	88 min	Baseline, and after follow up duration	Training with an interactive E-learning program	Routine educations	2 months	Skills	Mean score of delirium recognition ability in the intervention group significantly increased (*P* < 0.001), Mean score of delirium recognition ability in the control group did not significantly change (*P* = 0.055)	Interactive E-learning is effective in significantly improving critical care nurses’ delirium recognition ability and reducing their strain of care
Saiboon,2016, Malaysia [31]	Saudi medical journal	Ventricular fibrillation	80 nurses, Interventional group: 41 (31.32 ± 4.07) Control group: 39 (30.62 ± 5.37)	RCT	SLP teaching video	ICU	7 days,	Baseline, after intervention and after follow up duration	Training with SLP teaching video	Routine educations	6 months	knowledge, and skills	Among the participating nurses with 34 out of 37 (91.0%) in the TCI group compared with 31/34 (91.2%) in the SIV group able to successfully perform defibrillation immediately after exposure to the different teaching methods	SIV is as good as TCI in providing the knowledge, competency, and confidence in performing AED defibrillation
Yu,2021, South Korea [32]	Asian Nursing Research	High-risk neonatal infection control performance	50 Senior nursing students, Interventional group: 25 (22.44 ± .87) Control group: 25 (22.36 ± 1.22)	Quasi-experimental design	Virtual reality simulation	NICU	Three scenarios with 40 min	Baseline and after intervention	Training with Virtual Reality simulation program software	Routine educations	Without follow up	knowledge, attitude, and self-efficacy	Compared to the control group, the experimental group showed significantly improvements in high-risk neonatal infection control performance self-efficacy (t ¼ 2.16, p ¼ .018) and learner satisfaction (t ¼ 5.59, *p* < .001)	The virtual reality simulation program can expand the nursing students’ practice experience in safe virtual spaces and enhance their performance self-efficacy and learning satisfaction
Azizian,2020, Iran [33]	Journal of Education and Health Promotion	Performing endotracheal tube suctioning	44 nurses, Interventional group: 22 (33.14 ± 7.27) Control group: 22 (31.82 ± 8.29)	Quasi-experimental design	EV	ICU	Not mentioned	Baseline and after intervention	Training with educational video	Performance feedback	Without follow up	Performance (skills)	Before and after the intervention, a significant improvement was observed in the total mean score and other dimensions of nurses’ practice in endotracheal suctioning (*P* < 0.0001)	The results showed that both of methods through feedback and EV are useful in improving nurses’ ETS practice
McCutcheon,2018, UK [34]	International Journal of Nursing Studies	Clinical Supervisee Training	125 undergraduate final year nursing students, Intervention group: 62 Control group: 60	RCT	Clinical Supervisee Training app	ICU	30 min each session, 2 or 4 weeks	Only after intervention	Face-to-face tutorial and the online clinical supervisee skills training app	Clinical supervisee skills training app	Without follow up	knowledge, compassion and attitudes	The blended group had a more positive position on the scale of attitudes and this difference is statistically significant (*p* = .001). Participants in the blended group had a higher success rate in the knowledge test and this difference is statistically significant (*p* = .015). Participants in the blended group indicated a higher level of compassion and this difference is statistically significant (*p* = .001)	Blended learning provides added pedagogical value when compared to online learning in terms of teaching undergraduate nurses clinical supervision skills
Kes,2021, Turkey [35]	Nursing in critical care	Arrhythmia interpretation skills	66 nurses, Intervention group: 33 Control group: 33	RCT	SMS messaging via WhatsApp	ICU	During an 8-week period, twice a week (one on Tuesday and one on Saturday)	Baseline and follow-up (3 and 6 months)	Deliver SMS via WhatsApp	Without intervention	Without follow up	Skills	The analysis indicated that ICU nurses who received SMS messages about cardiac arrhythmias two times a week had significantly increased scores (*P* < 0.001)	This study concluded that using SMS messages as a training tool has a positive influence on cardiac arrhythmias interpretation skills among ICU nurses

*Abbreviations*: *ICU* Intensive Care Unit, *EOL* End-Of-Life, *RCT* Randomized Control Trial, *CPR* Cardiopulmonary Resuscitation, *CICU* Cardiac Intensive Care Unit, *TTM* Targeted Temperature Management, *NICU* Neonatal Intensive Care Unit, *SLP* Self-Learning Package, *EV* Educational Video, *AED* Automated External Defibrillator, *SMS* Short Message Service, *SIV* Self-Instruction Videos, *TCI* Traditional Classroom Instruction

Note that the study design was mostly experimental and of the Randomized Controlled Trial (RCT) type. Four studies (40%) were designed in the form of RCT, one study was in the form of a clustered randomized controlled trial, and finally, five studies were set in the form of quasi-experimental design (before and after with two experimental and control groups).

In seven studies, included participants were samples from Intensive Care Units (ICU) (*n* = 7, 70%); in one study, the participants' setting was Neonatal Intensive Care Unit (NICU) (*n* = 1, 10%), and again in two investigations, participants were included from Cardiac Intensive Care Unit (CICU) (*n* = 2, 20%).

In the included studies and experimental groups, different technology and innovative educational contexts were used; video-oriented learning and virtual reality-based environments were the most popular technologies applied to train the nurses. In three studies (*n* = 3, 30%), the educational environment was interactive videos, and in two studies (*n* = 2, 20%), virtual reality was employed with its appendices. It has also been used in two studies of mobile or Windows-based applications. The control group received routine training in six studies such as booklet, article, and guidelines. Note that there was a follow-up period in only four studies.

### Meta-analysis

**Fig. 5:**
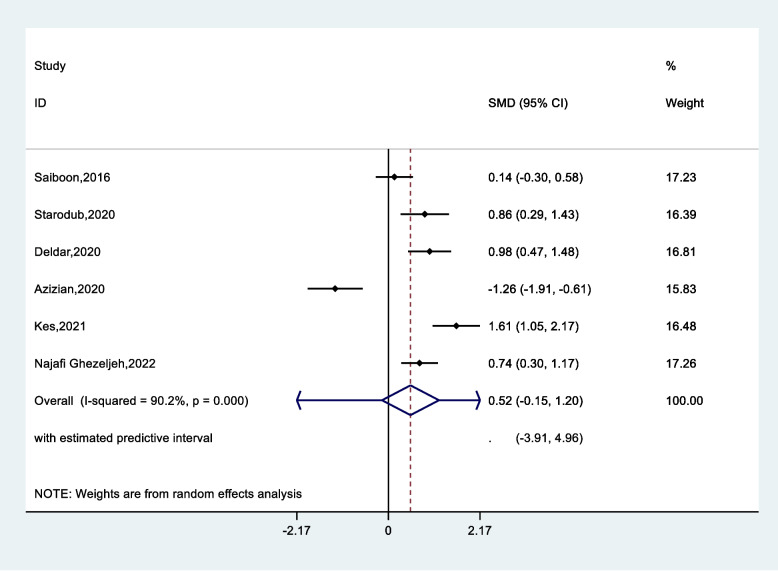
Pooled standard mean difference according to skill between experimental and control groups

**Fig. 6:**
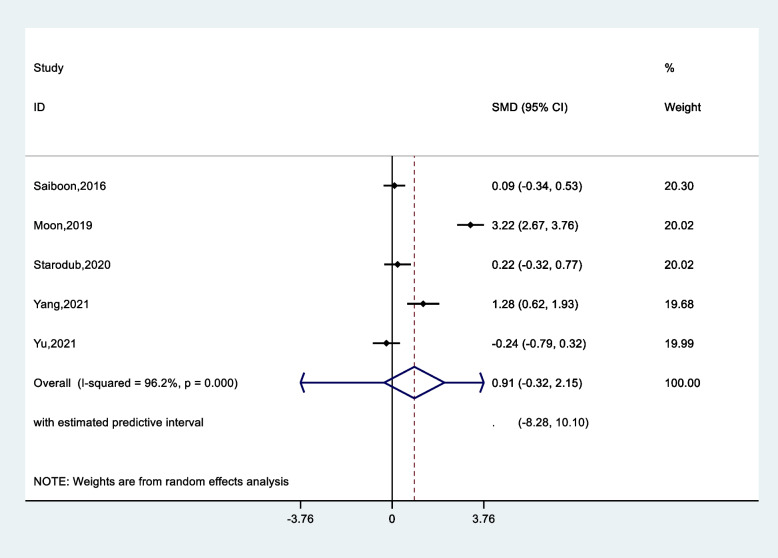
Pooled standard mean difference according to knowledge between experimental and control groups

Since one of the articles did not provide a pre-intervention evaluation, it was removed from the meta-analysis, and its results were presented only in our systematic review. The results of the meta-analysis are reported shown in Table 3. According to the results for skill parameter Standardized Mean Difference (SMD) was 0.52 (CI 95%, -0.15–1.20), suggesting that the standardized mean skill was 0.52 higher in the experimental education group than in the comparison group (Fig. 5). However, this difference was not significant (*p* = 0.13). Pooled effect size was higher for knowledge (SDM = 0.91, CI 95%, -0.32- 2.15 (*p* = 0.15)) (Fig. 6) and self-confidence (SDM = 0.96, CI 95%, -0.12- 2.06 (*p* = 0.08)) in the experimental group compared to the comparison group. In addition, in the analysis of subgroups, the pooled effect size of staff compared to students (0.50 vs. 1.49) and for self-confidence was estimated as (2.11 vs. 0.40). Eventually, less value was estimated for attention effect in the comparing groups.

**Table 3:** Pooled standard mean difference, heterogeneity and publication bias according nursing learning outcomes

**Parameters**	**Total**	**N. study**	**Pooled standard mean difference, (95%, CI)**	***p*-value (effect)**	**Heterogeneity test**		**Publication bias**	
					**I**^**2**^	**τ**^**2**^	**Egger’s**	**Begg’s**
**Skill**	Total	6	0.52 (-0.15- 1.20)	0.13	90.2	0.64	0.65	0.85
**Knowledge**	Total	5	0.91 (-0.32- 2.15)	0.15	96.1	1.9	0.59	0.62
	**Subgroup** **Nursing Staff** **Nursing Student**	3 2	0.50 (-0.21- 1.21) 1.49 (-1.83- 4.48)	0.57	81.2 98.7	0.32 5.88		
**Self confidence**	Total	3	0.96 (-0.12- 2.06)	0.08	91.9	0.83	0.23	0.12
	**Subgroup** **Nursing Staff** **Nursing Student**	1 2	2.11 (1.38- 2.84) 0.40 (-0.01- 0.81)	0.001	- 38.9	- 0.04		
**Attention**	Total	2	-0.32 (-1.7- 1.1)	0.65	95.7	0.95	0.11	0.09

### Risk of bias and heterogeneity

Additional analyses showed that no publication bias was observed in any of the analyses (*p* < 0.05). Regarding the true heterogeneity (**τ**^**2**^) among studies, in most variables it was very low (less than one).

### Limitation of studies

All mined papers reported some limitations and faced important challenges. The limitations mentioned in the studies are provided in Table 4.

**Table 4:** Reported limitations of included studies

Reported limitations	References
Limited sample size	[26], [28], [31], [33], [34]
Generalization of the findings is limited	[27], [28], [32], [33], [34], [35]
Short period of learning (Limited timeframe)	[26], [32]
Long-term effects of the study intervention were not measured	[30]
Impossibility of random allocation of individual participants	[30]
Analysis is limited to post-test	[34]
Setting inclusion criteria for sample size	[35]
Not blinding the participants to the intervention	[35]
Unawareness about the technical aspects participants ‘reluctance to use	[29]
The pre-knowledge levels for CPR in the two groups differed	[27]
The app was developed for Android operating system and has not yet be implemented on iOS devices	[26]

## Discussion

### Principal findings

The main intention of our systematic review and meta-analysis was to assess as well as screen the critical results related to the effectiveness of applying technology-based educational tools for the nurses and nursing students in ICUs. Accordingly, this study was conducted to examine the prior studies on the effects of technology-based learning for nursing to provide the primary data for evidence-based nursing research by assessing the objective use of the characteristics as well as the effects of learning tools through a meta-analysis. To our knowledge, this investigation chiefly focuses on determining experimental interventions using educational tools to train special functions/skills, knowledge, self-confidence, attention, etc.

Overall, the ten studies included were evaluated as good quality studies but had some risk of bias. The results concerning the risk of bias, especially performance bias (i.e., the blinding of participants and personnel), can partly be explained by the chosen evaluation policy. In studies with high bias risk, neither participants nor personnel was blinded, whereas, in studies with low risk, participants were not blinded, but staff and assessors were blinded. Also, we assessed the quality of methodological quality of the included studies.

Ten studies included in this systematic review were conducted after 2016. Further, 60% of them were performed in Korea and Iran. This shows that technology-based learning has been around since 2010, when innovative technologies began to be widely distributed. In addition, it is an inevitable result that many studies have been conducted in Korea, where technology-based learning infrastructures have already been established [36]. This study provides evidence that technology-based learning has beneficial effects on various learning outcomes, including knowledge acquisition, trust, and satisfaction with learning compared to traditional learning methods [13, 37].

Three studies had focused on nursing students including senior nursing students and undergraduate final year. Most studies had been done on nurses since nurses in ICUs need basic training, and their job sensitivity is very high. The findings of this study can provide guidance for nursing instructors, indicating that the use of technology-based educational tools is an effective solution to transfer students from the learning environment to clinical practice. In this systematic review, skills, knowledge, performance trust, learning attitude, and learning satisfaction were essential and practical criteria for technology-based learning intervention studies.

The present meta-analysis has shown that in most cases, the effect of the intervention on the skills, knowledge, and self-confidence of the participants was powerful and significant. However, the magnitude and direction of the effect of technology-based learning on learning outcomes seem highly situational [3, 36]. As a result, the impact of modern learning tools is likely to be influenced by many, possibly confounding, factors that differ across different learning methods, topics, and outcomes. In some studies, the tests were executed between the post-intervention scores of the intervention and control groups, rather than between the mean differences from baseline evaluation.

In this study, the results of technology-based educational tools consisted of six studies that had measured skills, followed by five studies evaluating knowledge; three studies had assessed self-confidence in performance and learning satisfaction. Eventually, two investigations had checked the learning attention. Also, in this study, case–control interventional studies were included, while single-group before-after studies were excluded from the review to minimize the heterogeneity of the studies. Remarkably, five studies were performed in a quasi-experimental design. Indeed, it seems that considering the characteristics of nursing research performed on nurses and nursing students, there are certain limitations for the full implementation of such cases since quasi-experimental studies are equivalent to randomized trials.

Scientifically speaking, researchers should try to reduce the bias of quasi-experimental research to determine the impact of technology-based learning tools accurately. Most previous studies did not provide information on the course and timing of the intervention. Thus, more efforts should be made to correct this issue in future research [27, 32]. In addition, blinding research participants was impractical since the use of technology-based tools by nurses or students could not be hidden, so blinding nurses and students are tough. The meta-analysis results revealed that there was an overall positive effect size for the target variables. Improved skills, knowledge, performance confidence (confidence), as well as attitude in studies were reported, and differences were significant. Nevertheless, there was no positive effect on nurses’/students' attention; this may be due to the few studies that had evaluated the attention variable.

The results of our study led to similar results to the findings of recent meta-analyses related to learning based on new technologies in nursing education. Based on the systematic literature review by Voutilainen A. et al. [38], the applied e-learning method was more effective than the conventional teaching methods; the new techniques had the potential to improve the learning outcome significantly. Another meta-analysis revealed that smartphone-based mobile learning could effectively improve nursing students' attitudes and that the use of these smartphones had also a significant positive effect on improving knowledge and skills [36]. Another study proved that simulation-based learning had moderate to substantial effects on enhancing knowledge acquisition, self-confidence, and learning satisfaction among undergraduate nursing students [39].

Nevertheless, the difference between our study and recent meta-analyses was that we looked at educating nurses and students who were gaining knowledge and skills in the ICU, and their timely intervention was critical [3, 40]. Technical, assessment, relational, and teamwork competencies are all required for optimal performance. ICU nurses monitor patients, administer medications, assist patients with basic needs, chart care, and respond to emergencies. Unlike some other nurses, their patients are often intubated and ventilated [41]. They must know the ins and outs of more equipment than nurses who practice in a lower-stakes environment. Also, they are highly trained and skilled safety–critical professionals working as part of a multidisciplinary team [9, 31].

Based on the results of this systematic study, the included studies had significant limitations and challenges that cannot be ignored. The most critical challenges included limited sample size, limited generalization of the findings, and a short period of learning (limited timeframe) to evaluate the effectiveness of educational tools.

### Strengths and limitations

This review has combined the results of risk of bias assessment (Cochrane tool) and meta-analysis. There have been strengths and weaknesses in this study. The strengths of the study are as follows: (1) applying an extensive search strategy to identify a large number of studies (3410 investigations), (2) conducting searches to retrieve studies in four important databases, including WOS, Scopus, Medline (through PubMed), and Embase, (3) reviewing and evaluating studies to extract data by five authors independently, (4) using comprehensive tools to evaluate the quality of included studies and to assess the risk of bias.

We have also encountered some limitations in this study. The difficulty of comparing studies is due to the heterogeneity of the results, so we interpreted outcomes with caution, and no generalization of the effects on nursing education seems appropriate. Also, book chapters, letters, non-English articles, and conference proceedings were excluded.

## Conclusion

This systematic review and meta-analysis highlighted improving nurses' and nursing students' knowledge, skills, self-confidence, and motivation to use educational tools based on innovative technologies. This study explained that technology-based training solutions such as virtual reality, simulation-based e-learning, social networks, etc., have significant potential to improve outcomes such as the specific knowledge and skills of nurses or nursing students in ICUs. Also, these tools will lead to the satisfaction of the target group and enhance patients' quality of life due to proper training of nurses. The effects of the interventions are strongly influenced by the time of the intervention. However, it can be suggested that if the learning method based on the new technologies tested is more effective than conventional teaching methods, they are likely to improve the learning outcome significantly.

## Supplementary Information

**Additional file 1: Table S1.** PRISMA 2020 Checklist.

## Data Availability

All data generated or analyzed during this study are included in this published article**.**
